# Comparison of Different Approaches to Define the Applicability Domain of QSAR Models

**DOI:** 10.3390/molecules17054791

**Published:** 2012-04-25

**Authors:** Faizan Sahigara, Kamel Mansouri, Davide Ballabio, Andrea Mauri, Viviana Consonni, Roberto Todeschini

**Affiliations:** Milano Chemometrics and QSAR Research Group, Department of Environmental Sciences, University of Milano-Bicocca, P.za della Scienza 1-20126 Milano, Italy; Email: faizan.sahigara@unimib.it (F.S.); kamel.mansouri@unimib.it (K.M.); davide.ballabio@unimib.it (D.B.); andrea.mauri@unimib.it (A.M.); viviana.consonni@unimib.it (V.C.)

**Keywords:** QSAR, model validation, Applicability Domain, interpolation space

## Abstract

One of the OECD principles for model validation requires defining the Applicability Domain (AD) for the QSAR models. This is important since the reliable predictions are generally limited to query chemicals structurally similar to the training compounds used to build the model. Therefore, characterization of interpolation space is significant in defining the AD and in this study some existing descriptor-based approaches performing this task are discussed and compared by implementing them on existing validated datasets from the literature. Algorithms adopted by different approaches allow defining the interpolation space in several ways, while defined thresholds contribute significantly to the extrapolations. For each dataset and approach implemented for this study, the comparison analysis was carried out by considering the model statistics and relative position of test set with respect to the training space.

## 1. Introduction

The quantitative relationship between chemical structures and their properties can be established mathematically by means of QSARs and thus, given that the structural information is available, QSAR models can be used theoretically to predict the properties for those chemicals [1]. Due to increasing application of such QSAR models, there had been rising concerns with respect to their predictions [2]. Derivation of QSAR models is based primarily on training sets which are structurally limited and thus their applicability to the query chemicals is limited. In other words, the model can provide more reliable prediction for the external compounds that fall within these structural limitations [3].

A new European legislation on chemicals—REACH (Registration, Evaluation, Authorization and restriction of Chemicals) came into force in 2007, which deals with risk assessment of chemicals for their safe use, thus contributing to the human health and environment [4]. This law allows and encourages the use of QSAR model predictions when the experimental data are not sufficiently available or as supplementary information, provided validity of the model is justified [5]. Five OECD principles for QSAR validation adopted in November 2004 are the requisites of any given model proposed for regulatory use and can be significant to demonstrate the validity of QSAR models, which is crucial for REACH implementation. 

According to these OECD principles, the QSAR model should have: (1) a defined end point; (2) an unambiguous algorithm; (3) a defined domain of applicability; (4) appropriate measures for goodness-of-fit, robustness and predictivity and (5) a mechanistic interpretation, if possible [6]. The principles, in general, provide user with all the essential information regarding end-point being predicted, model algorithm used, scope of the model and associated limitations, model performance and understanding of how the model descriptors are associated with predicted endpoint [5]. This paper primarily focuses on the third OECD principle that deals with defining the Applicability Domain (AD) of a QSAR model.

The principle of Applicability Domain requires users to define the model limitations with respect to its structural domain and response space. As discussed above, the reliable QSAR predictions are limited generally to the chemicals that are structurally similar to ones used to build that model [7,8,9]. The query chemicals that satisfy the scope of the model are considered as within the AD and classified as interpolated whereas the rest are extrapolations and thus, outside the AD. Reliability in a given model is higher for predictions falling within the AD and it is most likely to be unreliable for the extrapolations. This implies that the fourth OECD principle dealing with model accuracy is highly dependable on the model’s AD since here the chemical space associated with reliable predictions is identified. Molecular descriptors used to build the model also play a significant role in defining the AD. Thus, if a query chemical differs in terms of the structural limitations defined by the training set, it can be considered as an outlier for that chemical space.

Defining a model’s AD is essential in order to determine the subspace of chemical structures that could be predicted reliably. In other words, the degree of generalization of a predictive model depends on how broad the domain of applicability is. If the domain is too restricted, this implies the model is capable of giving reliable predictions only for limited chemical structures. Also, for regulatory purposes, like in REACH, it is essential for the user to provide all possible documentation about the model’s AD. This is beneficial for the user to see if the endpoint for the chemical structures under evaluation can be reliably predicted. Also, for the cases where several QSAR models are available for chemicals of interest, the knowledge of AD can be applied to compare how reliable the predictions could be for different models [1].

Characterization of the interpolation space is very significant to define the AD for a given QSAR model. Several AD approaches have been already proposed and primarily they all differ in the way how they characterize the interpolation space defined by the descriptors used. They can be classified into following four major categories based on the methodology used for interpolation space characterization in the model descriptor space: Range-based methods, Geometric methods, Distance-based methods and Probability Density Distribution based methods [1,2,3,4,5].

In this study, the above mentioned AD approaches are discussed and compared, focusing on the methodology used and criteria followed to consider a query structure to be within (or outside) the Applicability Domain. The major goal of this paper is to provide a detailed comparison of the results obtained, using these different AD approaches on some selected datasets. Two models from the CAESAR project, which predict the bioconcentration factor (BCF), were chosen as the case study [10,11]. Apart from their own test sets, an alternative test set from EPI Suite package BCFBAF v3.00 was chosen to facilitate further evaluation of AD approaches [12,13]. The number of test compounds considered outside AD for different approaches was calculated and the reliability of these results was further interpreted by analyzing both, the prediction statistics and the relative position of test compounds with respect to the training space. For all distance measures in this study, the pattern of test compounds considered outside the AD was understood by implementing the distance-based approaches with several threshold defining strategies that considered both, the distances of training compounds from their mean as well as the average distances of training compounds from their first 5 nearest neighbors. Finally, comparing the results derived with this analysis, most preferred thresholds for distance-based approaches were chosen for their overall comparison with other AD approaches. 

## 2. Applicability Domain Methods

The basis for interpolation is to predict the function value at a given point when the values at neighboring points are known. There are several descriptor based approaches by which the interpolation regions in multivariate space can be estimated for QSAR models. In a given *p*-dimensional descriptor space, estimations for new query chemicals are then obtained using the training data [1]. All the approaches used for this study were implemented using MATLAB [14] and are discussed briefly in this section informing their main features to define the interpolation space as well as the thresholds criterion used. 

### 2.1. Range-Based and Geometric Methods

These are considered as the simplest methods to characterize a model’s interpolation space.

#### 2.1.1. Bounding Box

This approach considers the range of individual descriptors used to build the model. Assuming a uniform distribution, resulting domain of applicability can be imagined as a Bounding Box which is a *p*-dimensional hyper-rectangle defined on the basis of maximum and minimum values of each descriptor used to build the model. The sides of this hyper-rectangle are parallel with respect to the coordinate axes. However, there are several drawbacks associated with this approach: since only descriptor ranges are taken into consideration, empty regions in the interpolation space cannot be identified and also the correlation between descriptors cannot be taken into account [1,2]. 

#### 2.1.2. PCA Bounding Box

PCA transforms the original data into a new coordinate system by the rotation of axes, such that the new axes are orthogonal to each other and aligned in the direction having maximum variance within the data. These new axes are called Principal Components (PCs) representing the maximum variance within the dataset [15]. A *M*-dimensional hyper-rectangle (where *M* is the number of significant components) is obtained similar to the previous approach by considering the projection of the molecules in the principal component space, however taking into account the maximum and minimum values for the PCs. The implementation of Bounding Box with PCA can overcome the problem of correlation between descriptors but empty regions within the interpolation space still remains an issue [1,2,5]. Moreover, selection of appropriate number of components is significant to implement this approach.

#### 2.1.3. Convex Hull

With this approach, interpolation space is defined by the smallest convex area containing the entire training set. Implementing a Convex Hull can be challenging with increasing data complexity [16]. For two or three dimensional data, several algorithms are proposed; however, increase in dimensions contribute to order of complexity. In addition, set boundaries are analyzed without considering the actual data distribution. Similar to the Range-based approaches, Convex Hull cannot identify the potential internal empty regions within the interpolation space [1,2].

### 2.2. Distance-Based Methods

These approaches calculate the distance of query compounds from a defined point within the descriptor space of the training data. The general idea is to compare distances measured between defined point and the dataset with a pre-defined threshold. The threshold is a user defined parameter and is set to maximize the separation of dense regions within the original data. However, the cut-off value does not entirely reflect the actual data density [1,2,3,4,5]. No strict rules were evident from the literature about defining thresholds for distance-based approaches and thus it is up to the user how to define them. In this study, for all the distance measures, several possible threshold defining strategies were considered, the derived results were compared and finally the appropriate thresholds were chosen to overall compare their results with the ones derived from Range-based, Geometric and Probability Density Distribution based approaches. Some commonly used and most useful distance measures in QSAR studies include Mahalanobis, Euclidean and City Block distances [2,5]. 

The unique feature associated with Mahalanobis measure is the co-variance matrix which can handle the correlated descriptors. The other two distance measures lack this characteristic and thus require an additional treatment; for example, PC rotation to correct for the correlated axes. Iso-distance contours constitute the regions having constant distance measures and generally their shape differs with approaches according to the distance measure considered, for example, ellipsoids for Mahalanobis and spherical in case of Euclidean distances [2]. 

Apart from them, similar approaches based on leverage are quite recommended for defining AD of a QSAR model [17]. Leverage of a query chemical is proportional to its Mahalanobis distance measure from the centroid of the training set. The leverages are calculated for a given dataset **X** by obtaining the leverage matrix (**H**) with the equation below:



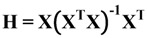
(1)


where **X** is the model matrix while **X**^T^ is its transpose matrix.

Diagonal values in the **H** matrix represent the leverage values for different points in a given dataset. Compounds far from the centroid will be associated with higher leverage and are considered to be influential in model building. Leverage is proportional to Hotellings T^2^ statistic and Mahalanobis distance measure but can be applied only on the regression models. The approach can be associated with a warning leverage, generally three times the average of the leverage that corresponds to *p/n* where *p* is the number of model parameters while *n* is the number of training compounds. A query chemical with leverage higher than the warning leverage can be associated with unreliable predictions. Such chemicals are outside the descriptor space and thus be considered outside the AD [1,2,5]. In this study, the corresponding Mahalanobis measures were used.

#### K nearest Neighbors Approach

This approach is based on providing similarity measure for a new chemical with respect to the compounds within the training space. The similarity is accessed by finding the distance of a query chemical from nearest training compound or its distances from *k* nearest neighbors in the training set. If these distance values are within the user defined threshold, the query chemical with higher similarity is indicated to have higher number of training neighbors and therefore, is considered to be reliably predicted. Thus, similarity to the training set molecules is significant for this approach in order to associate a query chemical with reliable prediction [9].

### 2.3. Probability Density Distribution-Based Method

Considered as one of the most advanced approaches for defining AD, these methods are based on estimating the Probability Density Function for the given data. This is feasible by both, parametric methods that assume standard distribution and non parametric methods which do not have any such assumptions concerning the data distribution. A main feature of these approaches is their ability to identify the internal empty regions. Moreover, if needed, the actual data distribution can be reflected by generating concave regions around the interpolation space borders [1,2]. 

Generally these approaches are implemented by estimating probability density of the dataset followed by identifying Highest Density Region that consists of a known fraction (given as user input) from the total probability mass [1].

Potential is created for each molecule in the training set such that it is highest for that molecule and decreases with distance. Once the potential is calculated for all the compounds, global potential is obtained by adding the individual potentials thus indicating the probability density [18,19]. 

There are several types of potential functions; however, for this study Gaussian function was considered. Given two molecules *x_i_* and *x_j_*, it can be determined as below:



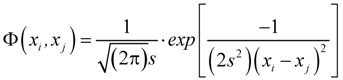
(2)


where 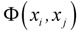 is the potential induced on *x_j_* by *x_i_* and width of the curve is defined by smoothing parameter *s*. The cut off value associated with Gaussian potential functions, namely *f_p_*, can be calculated by methods based on sample percentile [18]:


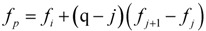
(3)


With 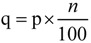, where p is the percentile value of probability density, *n* is the number of compounds in the training set and *j* is the nearest integer value of q. Test compounds with potential function values lower than this threshold are considered outside the AD.

### 2.4. Other AD Approaches

Apart from the AD strategies discussed above, several other approaches were published in literature to define the AD of QSAR models, some of which are briefly discussed below. These approaches were not considered for this comparative study since the analysis was limited to the classical AD methodologies used for interpolation space characterization in the model descriptor space. 

#### 2.4.1. Decision Trees and Decision Forests Approach

Based on the consensus prediction of Decision Trees (DT), this approach specifies the AD in terms of prediction confidence and domain extrapolation. The main idea here is to minimize the overfitting which can be achieved by combining the DTs and keeping the differences within different DTs to maximum possible. Predictions from all the combined DTs are averaged in order to determine the prediction confidence for a given compound while domain extrapolation provides the prediction accuracy for that compound outside the training space [1,20,21].

#### 2.4.2. Stepwise Approach to Determine Model’s AD

This approach is divided into four stages applied in a sequential manner. In the first stage, a query chemical is checked to fall within the range of variation of the physicochemical properties of training set compounds. During the second stage, structural similarity is found within the chemicals that are correctly predicted by the model. The third deals with mechanistic check while the reliability of simulated metabolism is taken into account in the final stage. To be considered within the AD, a query compound is required to satisfy all the conditions specified within these four stages. As a part of this rigorous approach, a chemical is evaluated for similarity, metabolic and mechanistic check, thus addressing the reliability of predictions and allowing a better assessment of model’s AD [3,5].

### 2.5. Models and Test Sets

This section deals with models and datasets selected for the comparison of the different AD approaches. 

#### 2.5.1. CAESAR Models

Bioconcentration factor, which is one of the most important endpoints for environmental fate of chemicals, was chosen for comparing the results derived from the different AD approaches considered in this study. As the procedure requires deep knowledge of the model and also information about its datasets and building methods, two already existing models to predict BCF were considered [10,11]. 

The QSAR models (Model 2 and Model 5) used in this study were the selected best two BCF models developed under the EU project CAESAR taking into account the REACH requirements [10]. These two models based on Radial Basis Function Neural Network (RBFNN) [22] were rebuilt, each with five descriptors that were calculated using Dragon 5.5 [23].The obtained statistics are summarized in Table 1.

**Table 1:** An overview of selected CAESAR models.

Model	Training set		Test set	
	*R^2^* ^(a)^	*RMSE* ^(b)^	*Q^2^* ^(c)^	*RMSEP* ^(d)^
1) Model 2	0.804	0.591	0.797	0.600
2) Model 5	0.810	0.581	0.774	0.634

^(a)^ Determination coefficient *R^2^*; ^(b)^ Root-mean-square error *RMSE*; ^(c)^ Predictive squared correlation coefficient *Q^2^*; ^(d)^ Root-mean-square error of prediction *RMSEP*.

#### 2.5.2. CAESAR and EPI Suite Test Sets

The CAESAR dataset consisted of 473 compounds, randomly divided into a training set of 378 compounds and a test set of 95 compounds, as explained in the original study [10]. The *Q*^2^ and *RMSEP* values for the test sets of CAESAR Model 2 and Model 5 are reported in Table 1.

For a better evaluation of AD approaches, in addition to the CAESAR test set, the validation set of the BCF model from EPI Suite package BCFBAF was selected as an additional test set [12,13]. This test set was comprised of 158 compounds, from which one compound was discarded due to structure inadequacy while other 49 compounds were not considered due to overlapping with the CAESAR training set compounds. 

## 3. Results and Discussion

For the AD approaches discussed earlier, general rules to define thresholds are discussed in the literature except for distance-based approaches. Thresholds can be defined in several ways for the distance-based approaches, thus resulting in an ambiguity over selection of appropriate thresholds for this study. As a result, before an overall comparison of results with different AD approaches could be performed, thresholds for distance-based approaches had to be finalized. 

To decide upon appropriate thresholds for distance-based approaches, several threshold defining strategies were implemented for the different distance measures considered in this study. All these strategies discussed below required calculating distances of training compounds from their centroid. To evaluate further possibilities, the study was extended implementing these strategies however considering average distance of each training compound from their first 5 nearest neighbors. Model statistics were recorded each time and the most appropriate distance based thresholds were then selected from above mentioned results for all distance measures considered in this study. Until this point, all the four categories of AD approaches were associated with appropriate thresholds and finally subjected to overall comparison of results.

The results were tabulated informing the model’s statistics for each AD approach on the compounds considered inside the applicability domain using the following parameters:

i) Number of test compounds considered outside the domain of applicability;
ii) Predictive squared correlation coefficient *Q*^2^ [24,25]:

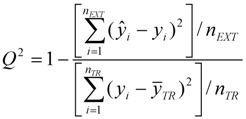
(4)


Where 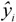 is the predicted value for the *i*-th compound and 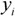 its experimental value; *n*_TR_ is the number of compounds in the training set and *n*_EXT_ the number in the test set; 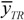 is the mean response of the training set. Moreover, in order to somehow quantify the role of the compounds considered inside and outside AD, 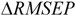 was defined by the following equation: 



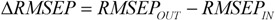
(5)


where *RMSEP_OUT_* is the root mean square error in prediction for the test compounds outside AD, while *RMSEP_IN_* is the root mean square error in prediction for the test compounds inside AD. Negative values indicate that the compounds detected outside AD are predicted better than the compounds inside AD, thus highlighting some possible drawbacks in the definition of interpolation space. On the contrary, positive values of 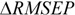 indicate a reliable partition for the compounds detected as inside and outside AD.

Multi Dimensional Scaling (MDS) was used to visualize the relative position of test compounds with respect to the training space. MDS enables the representation of *p*-dimensional data by means of a 2D plot. The implementation allowed a better understanding of how the interpolation space was characterized and if the compounds outside the AD were more concentrated around the training set extremities or not.

### 3.1. Defining Thresholds for Distance-Based AD Approaches

Initially, the distances of training compounds from their centroid were calculated and from this resulting vector, the maximum and average distance value (*maxdist* and *d*) were derived. The first threshold strategy defined the AD considering *maxdist* as threshold [2]. The second and third strategies considered twice and thrice the values of *d* as their thresholds, respectively. The fourth strategy performed percentile approach on the above derived vector of distances sorted in ascending order and the distance value corresponding to 95 percentile (*p95*) was chosen as the threshold. Finally, the fifth strategy (*dsz*) considered average distance *d* as well as the standard deviation from the distance vector (*std*) and the threshold was then defined as 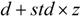, where *z* is the arbitrary parameter and is set to 0.5 as default value [26]. 

For all the cases, distance of a test compound from the training set centroid is compared with the defined threshold. If the distance of this test compound from the training set centroid is less than or equal to the threshold value, it is considered inside the AD. Thus, these approaches differ the way in which thresholds are derived, however the principle behind considering a given test compound to be inside or outside AD remains the same. Results derived with all the four threshold strategies are shown in Table 2 for CAESAR Model 2 considering different distance measures.

**Table 2:** Statistics for CAESAR Model 2 implementing distance-based approaches with different thresholds. For the acronyms *maxdist*, *d*, *p95*, *dsz*, and *ΔRMSEP*, refer to text.

*Approach*	*Thresholds*	*Compounds outside the AD*		*Q^2^*		*ΔRMSEP*	
		*CAESAR*	*EPI Suite*	*CAESAR*	*EPI Suite*	*CAESAR*	*EPI Suite*
		*out of 95 (%)*	*out of 108 (%)*				
Euclidean (*maxdist*)	0.942	0 (0.0)	4 (3.7)	0.797	0.703	-	1.436
Euclidean (3*d)	1.018	0 (0.0)	1 (0.9)	0.797	0.676	-	0
Euclidean (2*d)	0.679	7 (7.4)	12 (11.1)	0.802	0.718	0.146	0.753
Euclidean (*p95*)	0.663	7 (7.4)	12 (11.1)	0.802	0.718	0.146	0.753
Euclidean (*dsz*)	0.423	15 (15.8)	36 (33.3)	0.791	0.741	−0.064	0.381
CityBlock (*maxdist*)	1.472	0 (0.0)	1 (0.9)	0.797	0.676	-	2.713
CityBlock (3*d)	1.863	0 (0.0)	0 (0.0)	0.797	0.616	-	-
CityBlock (2*d)	1.242	3 (3.1)	6 (5.5)	0.804	0.699	0.267	−1.049
CityBlock (*p95*)	1.084	8 (8.4)	11 (10.1)	0.801	0.705	0.068	0.717
CityBlock (*dsz*)	0.748	18 (18.9)	38 (35.1)	0.786	0.739	−0.093	0.361
Mahalanobis (*maxdist*)	6.614	0 (0.0)	0 (0.0)	0.797	0.616	-	-
Mahalanobis (3*d)	6.027	0 (0.0)	0 (0.0)	0.797	0.616	-	-
Mahalanobis (2*d)	4.018	6 (6.3)	5 (4.6)	0.791	0.624	−0.174	0.162
Mahalanobis (*p95*)	4.034	6 (6.3)	5 (4.6)	0.791	0.624	−0.174	0.162
Mahalanobis (*dsz*)	2.497	21 (22.1)	27 (25.0)	0.778	0.706	−0.138	0.354

No test compounds emerged outside the AD with first two strategies considering CAESAR test set, due to the higher threshold values; however, comparing the model statistics with the other approaches, this probably implies some possible drawbacks of these strategies in defining the interpolation space. Comparable results were derived considering the third and fourth strategies which imply the thresholds corresponding to twice the value of *d* and that corresponding to 95 percentile converged significantly for both the test sets. Model statistics improved in most of the cases, thus reflecting a reasonable choice of compounds outside AD. The final strategy taking into account also the standard deviation provided the maximum number of test compounds outside the AD, however with no (or significant) improvement to the model statistics for both the test sets. A similar pattern was observed for compounds considered outside the AD with both the test sets, however, with respect to the number of compounds considered outside the AD with different threshold strategies, the values were comparatively higher with EPI Suite test set. This reflected how diverse both the test sets were in terms of their compounds and indicating that the CAESAR test set comprised of compounds more similar to the training data as compared to the other test set. None of the strategies performed well with Mahalanobis distance measure for CAESAR test set resulting in a negative *ΔRMSEP.* Similar pattern for compounds outside AD was observed for CAESAR model 5 and the corresponding results can be found in Table 3.

**Table 3:** Statistics for CAESAR Model 5 implementing distance-based approaches with different thresholds. *Maxdist*: Maximum distance between training compounds and centroid of the training set; *d*: Average distance of training compounds from their mean; *ΔRMSEP*: Difference between *RMSEP* for compounds outside and inside the AD.

*Approach*	*Thresholds*	*Compounds outside the AD*		*Q^2^*		*ΔRMSEP*	
		*CAESAR*	*EPI Suite*	*CAESAR*	*EPI Suite*	*CAESAR*	*EPI Suite*
		*out of 95 (%)*	*out of 108 (%)*				
Euclidean (*maxdist*)	0.942	0 (0.0)	2 (1.8)	0.774	0.647	-	0.598
Euclidean (3*d)	0.958	0 (0.0)	2 (1.8)	0.774	0.647	-	0.598
Euclidean (2* d)	0.639	3 (3.1)	9 (8.3)	0.783	0.665	0.329	0.354
Euclidean (*p95*)	0.614	4 (4.2)	11 (10.1)	0.783	0.673	0.266	0.367
Euclidean (*dsz*)	0.393	23 (24.2)	32 (29.6)	0.753	0.646	−0.128	0.044
CityBlock (*maxdist*)	1.472	0 (0.0)	2 (1.8)	0.774	0.647	-	0.598
CityBlock (3*d)	1.791	0 (0.0)	1 (0.9)	0.774	0.634	-	0.037
CityBlock (2*d)	1.194	1 (1.0)	5 (4.6)	0.772	0.657	−0.417	0.457
CityBlock (*p95*)	1.085	4 (4.2)	11 (10.1)	0.767	0.665	0.309	0.308
CityBlock (*dsz*)	0.723	21 (22.1)	32 (29.6)	0.751	0.639	−0.156	0.022
Mahalanobis (*maxdist*)	6.957	0 (0.0)	0 (0.0)	0.774	0.633	-	-
Mahalanobis (3*d)	6.121	0 (0.0)	0 (0.0)	0.774	0.633	-	-
Mahalanobis (2*d)	4.081	3 (3.1)	6 (5.5)	0.767	0.621	−0.445	−0.275
Mahalanobis (*p95*)	3.859	5 (5.2)	6 (5.5)	0.764	0.621	−0.327	−0.275
Mahalanobis (*dsz*)	2.495	23 (24.2)	18 (16.6)	0.760	0.637	−0.081	0.035

The study was further extended by implementing the above mentioned threshold strategies for each distance measure, but considering average distance of each training compound from its first 5 nearest neighbors. Given a *n* by *n* distance matrix where *n* is total number of training compounds, in all the cases, average distance of each training sample from its first five nearest training neighbors is found. Later, the gross average is derived from these average distance values which will be denoted henceforth as *D*. In the first and second case, twice and thrice the value of *D* is considered as threshold, respectively. For the third case, percentile approach discussed earlier in potential density distribution methods, is applied on the sorted average distances of all training compounds (used to calculate *D*) and the value corresponding to 95 percentile (*p95*) is considered as threshold [27]. For the last strategy (*DSZ*), besides calculating the gross average distance *D* from the first five nearest neighbors, also the standard deviation (*Std*) is calculated on the average distances. Finally, the threshold is defined as 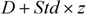, where *z* is the arbitrary parameter and is set to 0.5 as default value [26]. For all the cases, average distance of a test compound from its first five nearest neighbors in the training set is compared with the defined threshold. If the average distance for this test compound is less than or equal to the threshold value, it is considered inside the AD.

Results derived with all the four threshold strategies are shown in Table 4 and Table 5 for CAESAR Model 2 and Model 5, respectively, considering different distance measures. 

**Table 4:** Statistics for CAESAR Model 2 implementing different 5NN based threshold strategies. For the acronyms *D*, *p95*, *DSZ*, and *ΔRMSEP*, refer to text.

*Approach*	*Thresholds*	*Compounds outside the AD*		*Q^2^*		*ΔRMSEP*	
		*CAESAR*	*EPI Suite*	*CAESAR*	*EPI Suite*	*CAESAR*	*EPI Suite*
		out of 95(%)	out of 108(%)				
Euclidean (3*D)	1.522	2 (2.1)	1 (0.9)	0.804	0.676	0.394	2.713
Euclidean (2* D)	1.015	9 (9.5)	16 (14.8)	0.795	0.750	−0.037	0.765
Euclidean (*p95*)	1.164	8 (8.4)	13 (12.0)	0.797	0.745	0.859	1.342
Euclidean (*DSZ*)	0.693	14 (14.7)	31 (28.7)	0.787	0.767	−0.113	0.517
CityBlock (3*D)	2.371	4 (4.2)	5 (4.6)	0.803	0.679	0.187	0.968
CityBlock (2*D)	1.581	10 (10.5)	18 (16.7)	0.794	0.742	−0.042	0.664
CityBlock (*p95*)	1.918	7 (7.4)	11 (10.2)	0.799	0.741	0.034	0.944
CityBlock (*DSZ*)	1.083	16 (16.8)	27 (25.0)	0.801	0.731	0.037	0.446
Mahalanobis (3*D)	1.718	3 (3.2)	4 (3.7)	0.803	0.628	0.221	0.295
Mahalanobis (2*D)	1.145	9 (9.5)	18 (16.7)	0.794	0.748	−0.045	0.691
Mahalanobis (*p95*)	1.388	6 (6.3)	11 (10.2)	0.801	0.735	0.908	1.183
Mahalanobis (*DSZ*)	0.786	19 (20.0)	29 (26.9)	0.795	0.745	−0.019	0.470

**Table 5:** Statistics for CAESAR Model 5 implementing different 5NN based threshold strategies. D: The gross average distance of training set compounds from their 5NN; *ΔRMSEP*: Difference between *RMSEP* for compounds outside and inside the AD.

*Approach*	*Thresholds*	*Compounds outside the AD*		*Q^2^*		*ΔRMSEP*	
		*CAESAR*	*EPI Suite*	*CAESAR*	*EPI Suite*	*CAESAR*	*EPI Suite*
		out of 95 (%)	out of 108 (%)				
Euclidean (3*D)	1.681	0 (0.0)	2 (2.8)	0.774	0.644	-	0.364
Euclidean (2* D)	1.121	7 (7.4)	13 (12.0)	0.781	0.690	0.130	0.437
Euclidean (*p95*)	1.331	1 (1.0)	7 (6.5)	0.772	0.656	−0.331	0.126
Euclidean (*DSZ*)	0.782	18 (18.9)	22 (20.4)	0.784	0.743	0.072	0.512
CityBlock (3*D)	2.684	1 (1.1)	5 (4.6)	0.772	0.648	−0.456	0.307
CityBlock (2*D)	1.789	9 (9.5)	12 (11.1)	0.788	0.690	0.190	0.462
CityBlock (*p95*)	2.302	2 (2.1)	8 (7.4)	0.785	0.657	0.529	0.310
CityBlock (*DSZ*)	1.232	19 (20.0)	30 (27.8)	0.782	0.753	0.055	0.433
Mahalanobis (3*D)	2.006	0 (0.0)	4 (3.7)	0.774	0.624	−0.326	−0.149
Mahalanobis (2*D)	1.337	6 (6.3)	10 (9.3)	0.779	0.683	0.115	0.482
Mahalanobis (*p95*)	1.668	2 (2.1)	6 (5.6)	0.771	0.631	−0.193	−0.043
Mahalanobis (*DSZ*)	0.933	21 (22.1)	24 (22.2)	0.792	0.713	0.110	0.356

As obvious from Table 4, lowest number of test compounds were considered outside AD with the strategy considering 3*D as threshold. When the thresholds were lowered to 2*D, several other test compounds were considered outside the AD, however, the model performed worse with CAESAR test set. Same pattern was observed considering EPI Suite test set however, without lowering the model statistics and the number of test compounds outside the AD were comparatively higher in this case. Strategy taking into account also the standard deviation, was associated with the lowest threshold value thus, restricting the AD. Large number of compounds were considered outside the AD without improving the model statistics. The percentile approach considered reasonable number of test compounds outside AD without any major impact on the model statistics and the results were comparatively better with EPI Suite test set. Similar results and considerations were derived with CAESAR model 5. 

The next and the final step was to finalize upon one threshold strategy for distance-based approaches. All the four above mentioned strategies behaved differently depending on the distance measure considered. A strategy that improved the model statistics for one distance measure couldn’t have similar impact for another distance measure. This observation couldn’t allow an easy interpretation towards finalizing upon one strategy. However, considering improved model statistics with reasonable number of test compounds considered outside the AD, the percentile approach was a preferred choice. Moreover, when the methodologies for different AD methods were described earlier, Probability Density Distribution method reflected the statistical significance of defining percentiles. These considerations concluded finalizing upon the percentile approach for overall comparison of the results. This approach was implemented initially considering the distance of training compounds from their centroid (*p95*) and in the later case, based on average distance of training compounds from their 5 nearest neighbors (*p95*). Both the considerations were different in defining the interpolation space and thus, resulted in different number of compounds outside the AD with the same distance measure. Information derived in both the cases was significant and thus was retained for the overall comparison of the results.

### 3.2. Overall Comparisons

The distance-based approaches were then compared with other previously discussed AD approaches, considering the both CAESAR (95 compounds) and EPI suite (108 compounds) test sets. The results are summarized in Table 6 and Table 7 for CAESAR Model 2 and Model 5, respectively.

As shown in Table 6, by performing PCA analysis along with Bounding Box approach on Model 2, two test compounds were considered outside the AD. Convex Hull and Probability Density approach led to maximum number of test compounds outside the AD, thus decreasing the generalization ability of the models. *p95* approach lowered the model statistics for Mahalanobis distance measure. *Q*^2^ slightly lowered for Convex Hull that considered several test compounds outside the AD. On the other hand, model statistics improved for Probability Density Distribution approach which was associated with the maximum number of test compounds outside the AD (42.6%). As a general remark, the model statistics improved for several approaches with increase in number of test compounds considered outside the AD. Since the CAESAR test set comprised compounds more similar to the training set, not many test compounds emerged outside the AD; however, the EPI suite test set is comparatively different from the training data and thus considerably more compounds were outside the AD by different approaches. *ΔRMSEP* remained positive considering most of the AD approaches. Similar pattern for compounds outside the AD was derived for CAESAR model 5 and the corresponding results are reported in Table 7.

**Table 6:** Statistics for CAESAR Model 2 applied to CAESAR and EPI Suite test sets for different AD approaches.

*Approach*	*Compounds outside the AD*		*Q^2^*		*ΔRMSEP*	
	*CAESAR*	*EPI Suite*	*CAESAR*	*EPI Suite*	*CAESAR*	*EPI Suite*
	*out of 95 (%)*	*out of 108 (%)*				
Euclidean Dist. *(p95)*	7 (7.4)	12 (11.1)	0.802	0.718	0.146	0.753
City Block Dist. *(p95)*	8 (8.4)	11 (10.1)	0.801	0.705	0.068	0.717
Mahalanobis Dist. *(p95)*	6 (6.3)	5 (4.6)	0.791	0.624	−0.174	0.162
5NN-Euclidean Dist. (*p95*)	8 (8.4)	13 (12.0)	0.797	0.745	0.859	1.342
5NN-CityBlock Dist. (*p95*)	7 (7.4)	11 (10.2)	0.799	0.741	0.034	0.944
5NN-Mahalanobis Dist. (*p95*)	6 (6.3)	11 (10.2)	0.801	0.735	0.908	1.183
Bounding Box	0 (0.0)	2 (1.8)	0.797	0.678	-	1.798
PCA Bounding Box	2 (2.1)	3 (2.8)	0.804	0.688	0.371	1.533
Convex Hull	22 (23.2)	31 (28.7)	0.789	0.721	−0.052	0.368
Potential Function	29 (30.5)	46 (42.6)	0.831	0.766	0.156	0.374

**Table 7:** Statistics for CAESAR Model 5 applied to CAESAR and EPI Suite test sets for different AD approaches.

*Approach*	*Compounds outside the AD*		*Q^2^*		*ΔRMSEP*	
	*CAESAR*	*EPI Suite*	*CAESAR*	*EPI Suite*	*CAESAR*	*EPI Suite*
	*out of 95 (%)*	*out of 108 (%)*				
Euclidean Dist. *(p95)*	4 (4.2)	11 (10.1)	0.783	0.673	0.266	0.367
City Block Dist. *(p95)*	4 (4.2)	11 (10.1)	0.767	0.665	0.309	0.308
Mahalanobis Dist. *(p95)*	5 (5.2)	6 (5.5)	0.764	0.621	−0.327	−0.275
5NN-Euclidean Dist. (*p95*)	1 (1.0)	7 (6.5)	0.772	0.656	−0.331	0.126
5NN-CityBlock Dist. (*p95*)	2 (2.1)	8 (7.4)	0.785	0.657	0.529	0.310
5NN-Mahalanobis Dist. (*p95*)	2 (2.1)	6 (5.6)	0.771	0.631	−0.193	−0.043
Bounding Box	0 (0.0)	1 (0.9)	0.774	0.634	-	0.037
PCA Bounding Box	0 (0.0)	2 (1.8)	0.774	0.634	-	0.021
Convex Hull	16 (16.8)	21 (19.4)	0.780	0.643	0.049	0.051
Potential Function	28 (29.5)	47 (43.5)	0.787	0.813	0.062	0.455

To visualize where test set compounds were located with respect to the training compounds, multidimensional scaling (MDS) was performed. This enabled the representation of 5 dimensional data (the molecular descriptors defining the CAESAR models) by means of a two dimensional plot.

**Figure 1:**
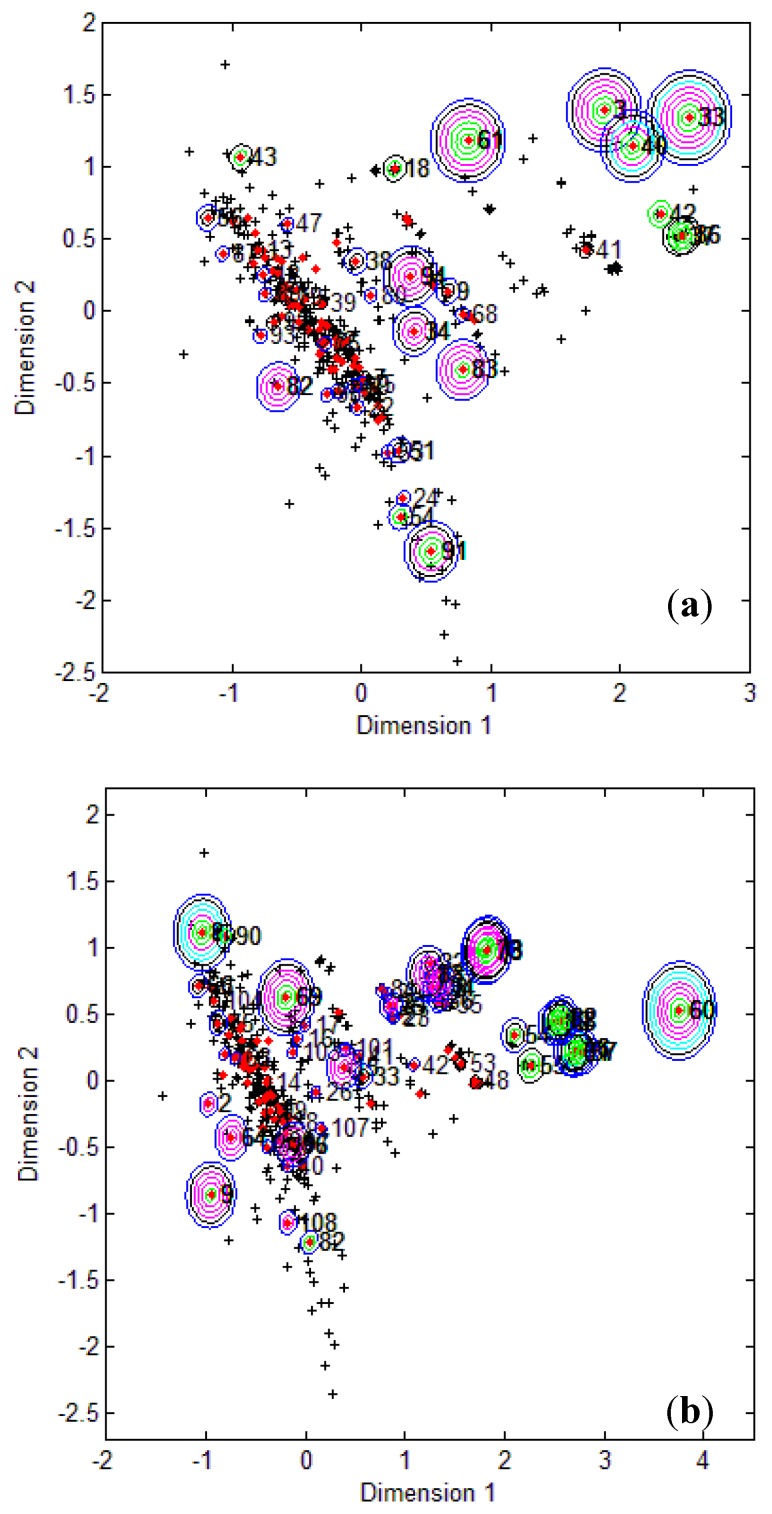
CAESAR test set (**a**) and Epi Suite test set (**b**) projected in the training space of Model 2. Training set (+); test set (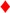); compounds outside the AD with different approaches; distance based *p95* (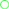), distance based 5NN (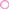), Bound. Box and PCA Bound. Box (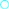), Conv. Hull (○), Pot. Funct. (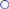).

**Figure 2:**
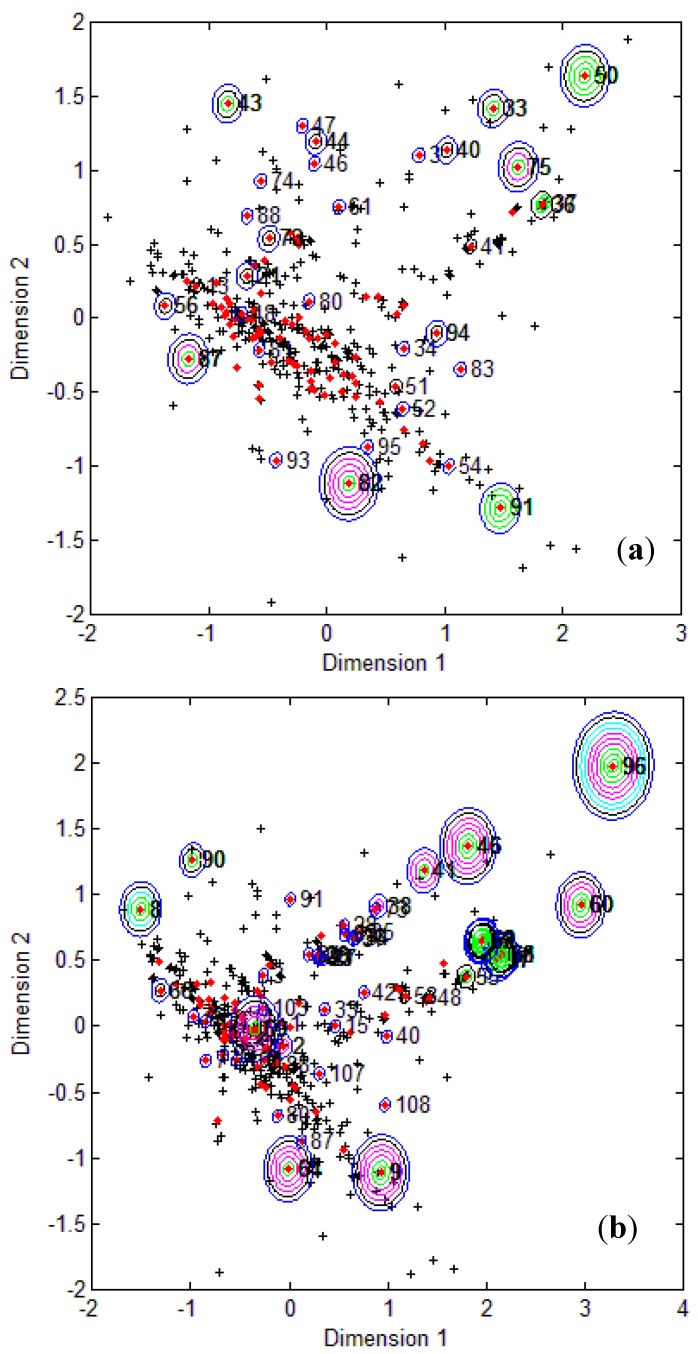
CAESAR test set (**a**) and Epi Suite test set (**b**) projected in the training space of Model 5. Training set (+); test set (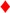); compounds outside the AD with different approaches; distance based *p95* (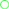), distance based 5NN (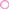), Bound. Box and PCA Bound. Box (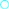), Conv. Hull (○), Pot. Funct. (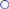).

From the MDS plots in Figure 1, it is clear that several test compounds that were localized towards the extremities of training set were considered outside the AD with most of the approaches. For example, CAESAR test compound 33 and EPI Suite test compound 60 were considered outside on the basis of 7 and 9 AD approaches, respectively. However, there were several compounds that were quite close to the training space but still falling outside the AD, especially with Convex Hull and Probability Density approaches (for example, CAESAR test compound 38 and EPI Suite test compound 33). Since the internal empty regions within chemical space cannot be easily detected and correlation between descriptors cannot be explained with Bounding Box, this approach failed to consider any test compound outside the AD. When the same approach was implemented on this dataset after PCA analysis, the correlation between descriptors was taken into account and as a result, two compounds from the test set were considered outside the AD. With respect to the EPI Suite test set, the MDS plots showed how most of test compounds outside the AD were lying in the training set extremities and were almost the same for different AD approaches. Those compounds were further more distant from training set than in the CAESAR test set. Similar results were derived for CAESAR model 5 and the corresponding plots are shown in Figure 2.

It was observed for both the CAESAR models that some compounds very close to the training compounds were considered outside the AD while others lying further were considered inside it. This could be explained by the fact that most of the implemented approaches considered only interpolation by simply excluding all test compounds in the extremities and including all those surrounded by training set compounds even if they are situated within empty regions of the chemical space.

**Figure 3:**
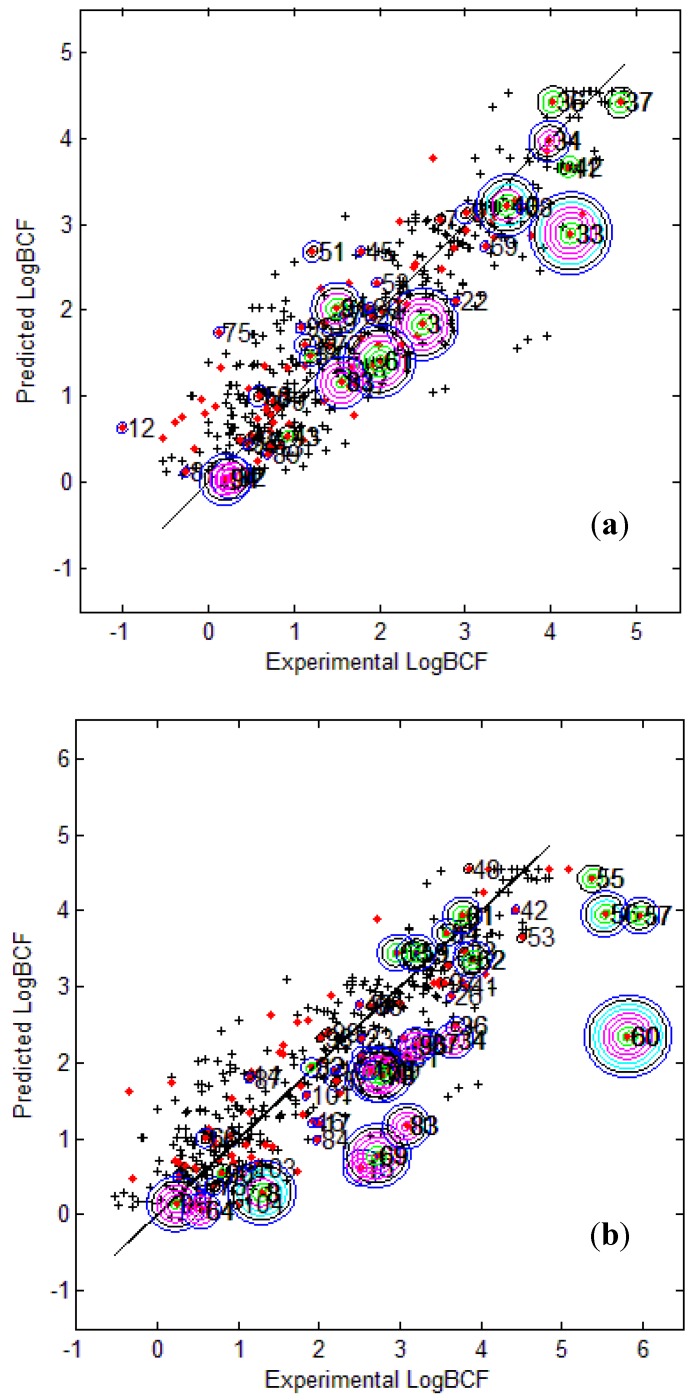
Predicted Vs observed log BCF values for CAESAR test set (**a**) and Epi Suite test set (**b**) with Model 2. Training set (+); test set (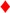); compounds outside the AD with different approaches; distance based *p95* (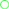), distance based 5NN (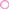), Bound. Box and PCA Bound. Box (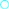), Conv. Hull (○), Pot. Funct. (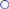).

**Figure 4:**
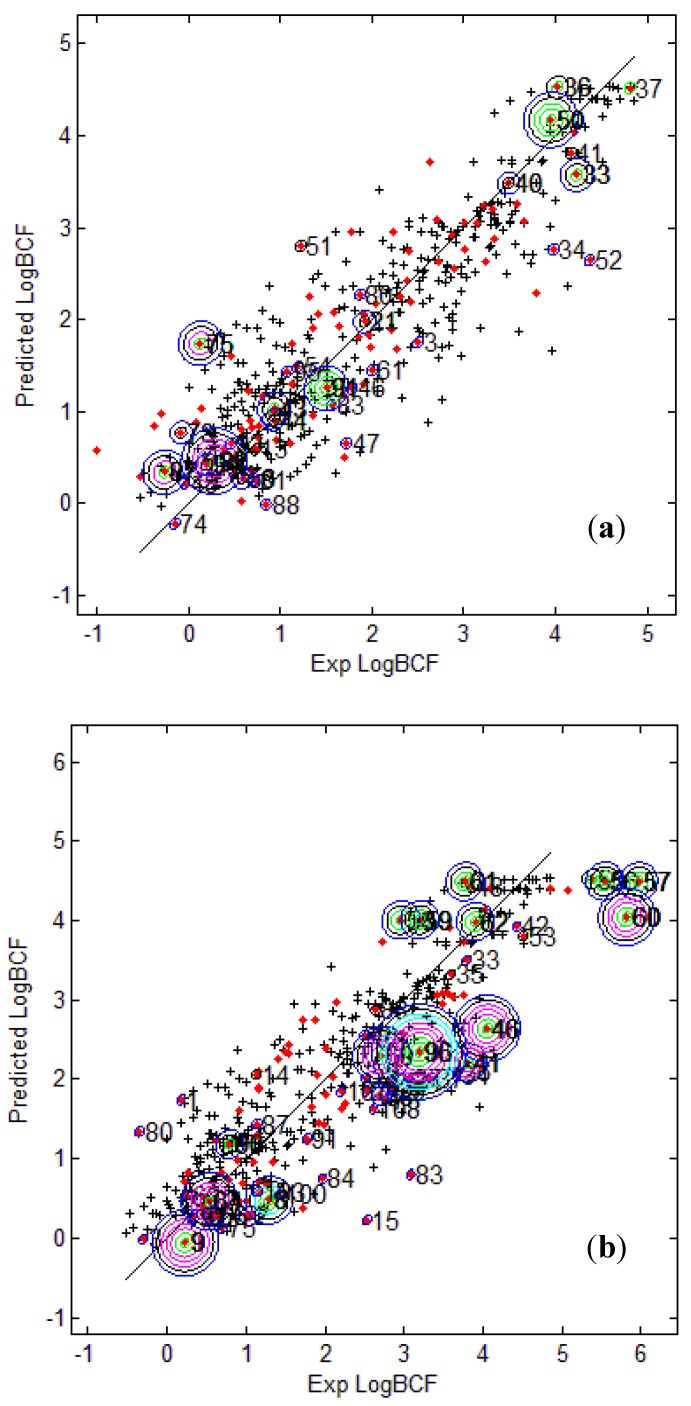
Predicted Vs observed log BCF values for CAESAR test set (**a**) and Epi Suite test set (**b**) with Model 5. Training set (+); test set (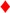); compounds outside the AD with different approaches; distance based *p95* (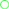), distance based 5NN (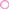), Bound. Box and PCA Bound. Box (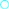), Conv. Hull (○), Pot. Funct. (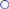).

Figure 3 provides the calculated logBCF values from the CAESAR Model 2 plotted against the experimental log BCF values (Exp logBCF). It can be noted that several test compounds not so reliably predicted were considered outside the AD. On the other hand, well predicted test compounds like 34 in CAESAR test set and 59 in EPI Suite test set were considered outside by 2 and 5 AD approaches respectively. This indicates that the strategy used by different AD approaches might have considered some well predicted compounds outside the AD, thus affecting the model statistics. As seen earlier in Table 6 and Table 7, Convex Hull and Probability Density Distribution approaches had considerable number of test compounds outside the AD; however, both the approaches differed significantly with respect to the model statistics. The results corresponding to CAESAR model 5 are plotted in Figure 4.

The plots indicate that several test compounds unreliably predicted were localized on the extremities of the training space and considered outside the AD while several well predicted test compounds were also considered outside with different approaches. This observation holds true for both the test sets however, the number of test compounds considered outside the AD were considerably higher for EPI Suite test set. Figure 3b shows that the three compounds 56, 57 and 60 considered outside the AD by several approaches were underestimated, and thus the model statistics highly improved with AD approaches not considering them within the domain of applicability. 

## 4. Conclusions

The characterization of interpolation space varied depending on the Applicability Domain approach implemented. Approaches compared in this study suffered from several limitations, some concerning the complexity of algorithm while some related to the algorithm used for defining interpolation space. Addition of PCA did not contribute significantly to the Bounding Box approach with the first test set however, with respect to the second validation set, performing PCA analysis had a significant impact on improving the model statistics. Probability Density Distribution approach and Convex Hull were associated with the highest number of test compounds outside the AD and thus allowing only a limited use of the models. Distance-based approaches considered reasonable number of test compounds outside the AD, however model statistics lowered for some distance measures. As expected, most of the test compounds considered outside the AD with most of the approaches were concentrated towards the training set extremities. It was clearly evident from the MDS plots that the distance from training space was significant in defining the model’s AD. Also, several test compounds badly predicted by the model were considered as outside the AD with most of the approaches. The results from the alternative test set provided were similar; however, number of test compounds outside the AD increased. When various thresholds were subjected to distance-based approaches, it was noted, however with some exceptions, that increase in the number of test compounds outside AD also improved the model’s statistics. Finally, all the implemented AD approaches had their own strengths and limitations and thus, it is up to the model builder to choose most appropriate applicability domain approach for his model. For instance, in this study, one of the aspects considered to evaluate a given AD approach was the number of test compounds outside the AD and its resulting impact on the model performance. It is important to note that the results derived with different AD approaches may vary for the same dataset and none of these approaches can be considered sufficient enough to be applied to all the cases; therefore, considering the present state of the art, it would be preferable to evaluate the results from all possible strategies before assessing a new compound set. 
